# Distinctive features of First Episode of Psychosis during the Covid-19 pandemic

**DOI:** 10.1192/j.eurpsy.2022.1330

**Published:** 2022-09-01

**Authors:** F. Casanovas, F. Dinamarca, A. Pérez Oms, C. Llimona Sánchez, D. García Hernández, J.M. Ginés, V. Pérez-Solà, D. Bergé, A. Mané

**Affiliations:** 1 Parc de Salut Mar, Institut De Neuropsiquiatria I Addiccions (inad), Barcelona, Spain; 2 Hospital del Mar Medical Institute, Imim, Barcelona, Spain

**Keywords:** brief psychotic disorder, Covid-19, Psychosis, Cannabis

## Abstract

**Introduction:**

COVID19 has brought several psychosocial stressors that are having an impact on global mental health. The impact of the pandemic on the incidence of First Episode of Psychosis (FEP) is not clear.

**Objectives:**

To describe the clinical and sociodemographic characteristics of FEP patients diagnosed since the onset of the COVID19 pandemic and compare them with the equivalent period of the previous year.

**Methods:**

We included all FEP patients attended at Parc de Salut Mar (Barcelona, Spain) from March 14, 2020 (when the state of emergency in Spain began) to December 31, 2020 with the same period of 2019. We assessed sociodemographic variables, duration of untreated psychosis (DUP), cannabis and alcohol use, psychiatric diagnosis, and psychiatric symptom scales. We performed a univariate analysis between the groups using U-Mann Whitney for continuous variables and Chi-Square for qualitative variables.

**Results:**

A total of 20 FEP patients were diagnosed in each period. No differences were found in sociodemographic variables, scales scores or DUP. During COVID19 period there was a smaller proportion of cannabis users (60% vs 90%; p=0.028) and a tendency of lower weekly consumption (14.44 vs 16.42; p=0.096). There were more cases of BPD (25% vs 5%; p=0.077) and less of affective psychosis (0% vs 25%; 0.017).

**Conclusions:**

During the COVID-19 pandemic we did not find an increase of FEP or more severe clinical presentations. However, we identified differences in the type of FEP that could be related to the psychosocial stressors of this time.

**Disclosure:**

No significant relationships.

